# The COOL-AF Phase 2 Registry: COhort of Antithrombotic Use and Clinical Outcomes in Atrial Fibrillation Patients

**DOI:** 10.1016/j.jacasi.2024.10.027

**Published:** 2025-01-21

**Authors:** Rungroj Krittayaphong, Arjbordin Winijkul, Voravut Rungpradubvong, Sirin Apiyasawat, Arintaya Phrommintikul, Thoranis Chantrarat, Komsing Methavigul, Ply Chichareon, Pattarapong Makarawate, Wattana Wongtheptien, Yodying Kaolawanich, Gregory Y.H. Lip

**Affiliations:** a Faculty of Medicine Siriraj Hospital, Mahidol University, Bangkok, Thailand; b Faculty of Medicine, Chulalongkorn University, Bangkok, Thailand; c Faculty of Medicine Ramathibodi Hospital, Mahidol University, Bangkok, Thailand; d Faculty of Medicine, Chiang Mai University, Chiang Mai, Thailand; e Phramongkutklao College of Medicine, Bangkok, Thailand; f Central Chest Institute of Thailand, Nonthaburi, Thailand; g Faculty of Medicine, Prince of Songkla University, Songkhla, Thailand; h Faculty of Medicine, Khon Kaen University, Khon Kaen, Thailand; i Chiangrai Prachanukroh Hospital, Chiangrai, Thailand; j Liverpool Centre for Cardiovascular Science at University of Liverpool, Liverpool John Moores University and Liverpool Heart and Chest Hospital, Liverpool, United Kingdom; k Danish Center for Health Services Research, Department of Clinical Medicine, Aalborg University, Aalborg, Denmark

**Keywords:** atrial fibrillation, ischemic stroke, oral anticoagulant

## Abstract

**Background:**

Atrial fibrillation (AF) is a common condition leading to an increased risk of death and complications such as stroke. Even though direct oral anticoagulants (DOACs) can reduce the risk of ICH, the rate of DOAC use remains low in many Asian countries because of cost concerns.

**Objectives:**

The purpose of this protocol paper of the COOL-AF (COhort of antithrOmbotic use and cLinical outcomes in patients with Atrial Fibrillation) Phase 2 registry is to determine the rate of clinical outcome, changes in antithrombotic patterns, and their impact on clinical outcomes, and to develop a prediction model for clinical outcomes.

**Methods:**

The COOL-AF Phase 2 study is a prospective observational multicenter study of patients with known or newly diagnosed nonvalvular AF in Thailand. The aim is to achieve a sample size of 3,667 patients from 33 centers. Patients will be followed up every 6 months for up to 3 years. Data collection on and doses of oral anticoagulants (warfarin, dabigatran, rivaroxaban, apixaban, and edoxaban) and antiplatelets are collected. The study outcomes include death, ischemic stroke/systemic embolism, major bleeding, myocardial infarction, heart failure, and quality of life. All events will be adjudicated.

**Results:**

Enrollment started in June 2024. The results of the COOL-AF phase 2 registry will be reported when enrollment is complete and one year of follow-up data is available.

**Conclusions:**

The COOL-AF Phase 2 trial will provide valuable information about the real-world practice of AF management and outcomes in Asia, which should be able to improve AF outcomes in the future. (COhort of antithrOmbotic Use and cLinical Outcomes in Patients With Atrial Fibrillation [COOL-AF] Phase 2; NCT06396299)

Atrial fibrillation (AF) is a common heart rhythm disorder that tends to increase in prevalence with advancing age.^1^ Besides its high global prevalence, AF also poses a significant health and financial burden.^2^ Patients with AF have approximately a 5% annual risk of developing ischemic stroke, which is about 5 times higher than that of individuals without AF.^3^^,^^4^

Most AF patients should receive stroke prevention with oral anticoagulants (OACs).^5^^,^^6^ Warfarin remains the primary OAC used in many Asian countries^5^^,^^7^ despite its disadvantages, including an increased risk of intracranial hemorrhage (ICH), a narrow therapeutic window, interacting with a wide range of foods and drugs, and a slow onset and offset of action.^8^ It is essential to monitor the international normalized ratio (INR) through blood tests to assess the medication's effectiveness.^9^ The dosage of the medication varies and is challenging to predict for each patient. Genetic factors that influence drug levels, and preliminary data indicate that the Asian population differs from Western countries,^10^ with a higher risk of ICH.^11^

Currently, there is a global shift in the guidelines for anticoagulant therapy, including in Thailand, where the use of direct oral anticoagulants (DOACs) for preventing ischemic stroke has become increasingly widespread.^12^, ^13^, ^14^ This trend is caused by their convenience of use, the lack of need for blood monitoring, and a reduced risk of bleeding, especially ICH, compared with warfarin. However, there are still limitations in accessing these medications in many low- or middle-income countries.^15^ Currently, DOACs are not included in the Thai National List of Essential Drugs because they are considered expensive drugs. A cost-utility analysis study in Thailand showed that all DOACs are not cost-effective at the current willingness to pay level in Thailand.^16^ It is recommended that DOACs be included in the World Health Organization Essential Medicines List.^17^ This is related to the low willingness to pay level in Thailand per quality-adjusted life year.^18^ It is uncertain whether the results from clinical trials can be applied in a real-world setting. The COOL-AF (COhort of antithrOmbotic use and cLinical outcomes in patients with Atrial Fibrillation) Phase 2 trial was initiated and funded by the Health Systems Research Institute and the Heart Association of Thailand under Royal Patronage. We intend to retain the acronym of the COOL-AF registry but with a different title because the original COOL-AF was planned to determine the optimal INR levels for those on warfarin. The COOL-AF Phase 2 aims to determine the clinical outcome of AF patients and to investigate contemporary antithrombotic treatment patterns for stroke prevention in patients with AF in Thailand and their impact on clinical outcomes. The primary objectives were as follows: 1) to determine the rate of clinical outcomes (death, SSE, and major bleeding); 2) to develop the prediction model for clinical outcomes; and 3) to determine the antithrombotic pattern and the effect on clinical outcomes. The secondary objectives were as follows: 1) to study the predictors for clinical outcomes; 2) to determine the effect of DOAC dosing on clinical outcomes, and to determine the effect of TTR on clinical outcomes in patients with warfarin; and 3) to validate model from COOL-AF phase 1, and to study economic analysis (Table 1).

**Table 1:** The Study Objectives

Primary objectives
To study the patterns of antithrombotic medication usage in patients with atrial fibrillation
To investigate the clinical outcome rates (including all-cause mortality, ischemic stroke/transient ischemic attack/systemic embolism, intracranial hemorrhage, and major bleeding) in atrial fibrillation patients
To develop predictive model for clinical outcomes
Secondary objectives
To investigate the factors predicting clinical outcomes in patients with atrial fibrillation;
To study the clinical outcome rates (including all-cause mortality, ischemic stroke/transient ischemic attack/systemic embolism, intracranial hemorrhage, and major bleeding) in atrial fibrillation patients stratified by different types of antithrombotic medications, including warfarin, stratified by TTR, the 4 types of DOACs, stratified by dosage, and antiplatelet agents;
To validate data from COOL-AF Phase 1, such as the COOL-AF predictive models for all-cause mortality, ischemic stroke/systemic embolism, and major bleeding; and
To conduct an economic analysis of the complications related to ischemic stroke and the cost-effectiveness of stroke prevention and treatment in atrial fibrillation patients.

COOL-AF = COhort of antithrOmbotic use and cLinical outcomes in patients with Atrial Fibrillation; DOAC = direct oral anticoagulant; TTR = time in therapeutic range.

## Methods

### Study design

**Figure 1:**
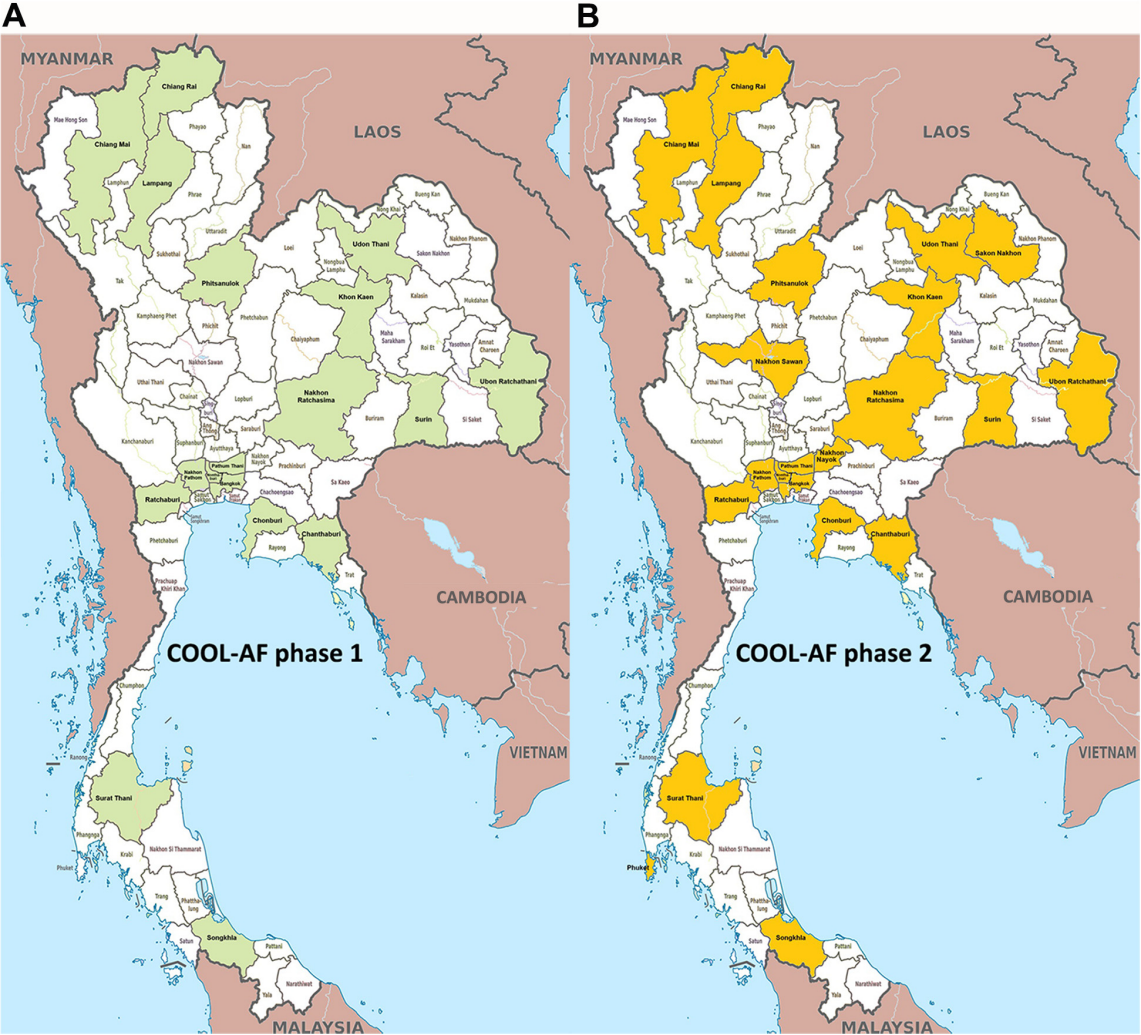
Study Sites for COOL-AF Phase 1 and 2 Registries This figure shows the locations of hospitals participating in the COOL-AF (COhort of antithrOmbotic use and cLinical outcomes in patients with Atrial Fibrillation) registry. (A) 27 hospitals involved in Phase 1, (B) 33 hospitals participating in Phase 2. The expansion in Phase 2 provides wider coverage across Thailand, allowing for better data collection on antithrombotic use and clinical outcomes in patients with atrial fibrillation.

COOL-AF Phase 2 is a prospective multicenter observational study for men and women older than 18 years with known or newly diagnosed AF documented by standard 12-lead ECG or Holter monitoring. We expect to enroll 3,667 patients from 33 centers distributed across different regions of Thailand (Figure 1).

The study was approved by the Central Research Ethics Committee (CREC) (COA-CREC103/2023). The methodology of this study was in accordance with the principles set forth in the Declaration of Helsinki and the International Conference on Harmonization for Good Clinical Practice Guidelines. All patients will provide written informed consent before participation. Participants have the right to withdraw their consent at any time after the enrollment.

### Study population

We studied patients with nonvalvular AF. Inclusion and exclusion criteria are shown in Table 2. The decision for enrollment depends on the judgment of the physician after consideration of the study criteria. To avoid bias, investigators were instructed to ensure consecutive patient enrollment. All required data will be recorded in the electronic web-based system.

**Table 2:** Inclusion and Exclusion Criteria of the Study Population

Inclusion Criteria	Exclusion Criteria
1. Participants age 18 y and older with atrial fibrillation diagnosed based on a 12-lead ECG or ECG tracing from ambulatory ECG monitoring 2. Participants must be willing to provide written informed consent to participate in the study.	1. Patients who have experienced an ischemic stroke within the past 3 mo before enrollment. 2. Patients with a platelet count of <100,000/mm^3^ or who have myeloproliferative disorders (essential thrombocythemia, chronic myeloid leukemia, polycythemia vera, agnogenic myeloid metaplasia), hyperviscosity syndrome, chronic disseminated intravascular coagulation, or antiphospholipid syndrome. 3. Patients with a mechanical prosthetic heart valve. 4. Patients with rheumatic mitral stenosis. 5. Patients participating in research projects with concealed treatments. 6. Patients expected to have a life expectancy of <3 y because of other diseases, such as cancer or acquired immune deficiency syndrome, as determined from medical records. 7. Pregnant patients. 8. Patients unable to follow the treatment plan. 9. Patients who have been hospitalized or discharged from the hospital within the past 1 mo.

ECG = electrocardiogram.

Patients will be identified from various sources such as the anticoagulation clinic, cardiology clinic, internal medicine clinic, geriatric clinic, and emergency department.

### Study protocol

After the consent process, investigators will record the required data from medical records and patient interviews. Patient data will be recorded in the web-based system that was created to be user-friendly for data entry. After the data entry is completed, the site investigators will submit the data.

**Figure 2:**
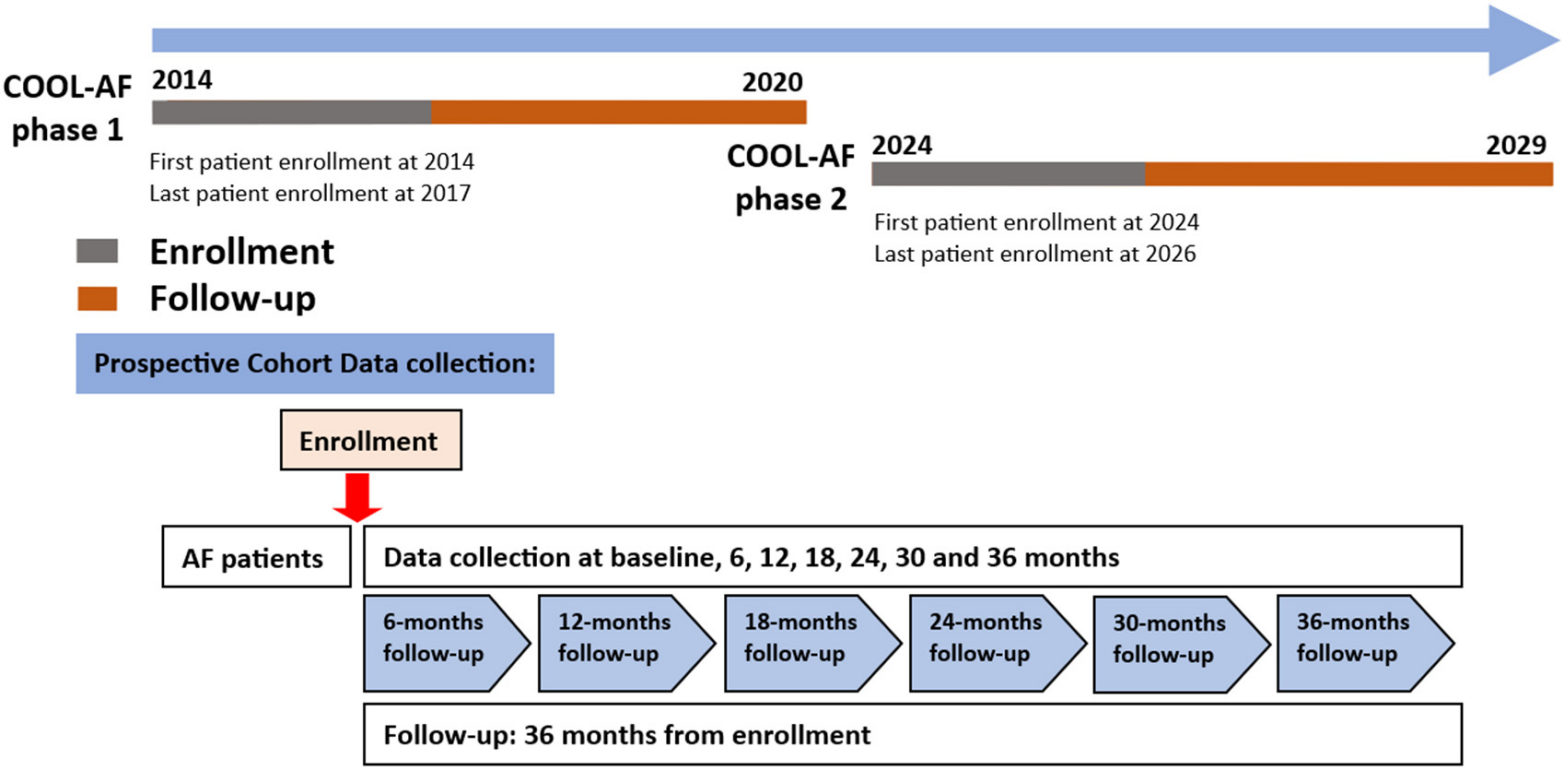
Enrollment and Follow-Up Timeline for COOL-AF Phase 2 This figure shows the timeline for the COOL-AF (COhort of antithrOmbotic use and cLinical outcomes in patients with Atrial Fibrillation) Phase 2 registry, starting with the first patient enrollment in June 2024. The last patient is expected in 2026. Follow-up assessments will take place at 6, 12, 18, 24, 30, and 36 months for each patient. The key clinical outcomes are tracked throughout the study. These outcomes include all-cause death, ischemic stroke, systemic embolism, major bleeding, myocardial infarction, heart failure, and quality of life.

Follow-up visit data will be recorded at 6, 12, 18, 24, 30, and 36 months after enrollment. The follow-up data will be recorded in a similar manner. The window period of data recording will be 1 month. Email reminders will be sent out to site investigators every 2 weeks to remind them of any overdue of the data entry or the incomplete record. The timeline of the study is shown in Figure 2.

### Data collection

The following data were collected at the baseline visit:

• Demographics (age, sex, weight, height)
• Type of AF (new, paroxysmal, persistent, permanent)
• Symptoms of AF (dyspnea, palpitations, dizziness, chest pain, syncope, fatigue, and the European Heart Rhythm Association score)
• Duration of AF
• Method of AF diagnosis
• Medical history (history of stroke/transient ischemic attack [TIA], carotid surgery/stenting, percutaneous coronary intervention, coronary artery bypass surgery, myocardial infarction, peripheral arterial disease, bleeding history, heart failure)
• Cardiovascular risk factors (hypertension, type 2 diabetes (T2D), smoking, dyslipidemia]
• AF management (rate control, rhythm control, cardioversion, ablation)
• Anticoagulant/antiplatelet therapy
• Vital signs (blood pressure, heart rate)
• INR values and dates
• Other laboratory values
• Cardiac imaging results
• Components of the CHA_2_DS_2-_VASc score
• Components of the HAS-BLED score
• Components of the SAMe-TT2R2 score
• EQ-5D-5L

Because antithrombotic pattern is one of the main interests of this registry, the data collection on the antithrombotic agents included the types and doses of OAC and antiplatelets. Anticoagulants are vitamin K antagonist (warfarin), and DOACs (dabigatran, rivaroxaban, apixaban, and edoxaban). Doses of DOACs are recorded as follows: dabigatran 110 and 150 mg twice daily, rivaroxaban 10, 15, or 20 mg once daily, apixaban 2.5 or 5 mg bid, and edoxaban 15, 30, or 60 mg od. Antiplatelets are aspirin and P2Y12 inhibitors (clopidogrel, prasugrel, and ticagrelor). Doses of aspirin are recorded. Combination of OAC and antiplatelet is also recorded. Data collection at the follow-up visit was similar to that at the baseline visit with the addition of clinical events and data related to that event. The source document of the clinical outcomes was required to be uploaded into the web system.

Schedule of study procedures at baseline and follow-up is shown in Table 3. Summary of study protocol and design of the COOL-AF Phase 2 study is shown in the Central Illustration.

**Table 3:** Schedule of Study Procedures

	Initial Visit	Data Collection Interval					
		6 mo	12 mo	18 mo	24 mo	30 mo	36 mo
Assessments (prescreen)							
Informed consent	√						
Inclusion/exclusion criteria	√						
Demographics	√						
Diagnosis	√						
Symptoms of AF	√	√	√	√	√	√	√
Cardiovascular history	√						
Additional medical history	√						
Bleeding history and risk factors for bleeding							
Vital signs	√	√	√	√	√	√	√
Investigations							
Laboratory INR values	√	√	√	√	√	√	√
Other laboratory findings	√	√	√	√	√	√	√
Cardiac imaging	√	√	√	√	√	√	√
AF treatment							
Nonmedication treatment	√	√	√	√	√	√	√
Rate and rhythm control medication	√	√	√	√	√	√	√
Antithrombotic therapies	√	√	√	√	√	√	√
Other medications	√	√	√	√	√	√	√
Components of CHA_2_DS_2_-VASc score	√	√	√	√	√	√	√
Components of HAS-BLED score	√	√	√	√	√	√	√
Components of SAMe-TT2R2 score	√	√	√	√	√	√	√
Outcomes		√	√	√	√	√	√
Quality of life (QOL) questionnaires							
EQ5D-5L	√				√		√

AF = atrial fibrillation, INR = international normalized ratio.

**Central Illustration:**
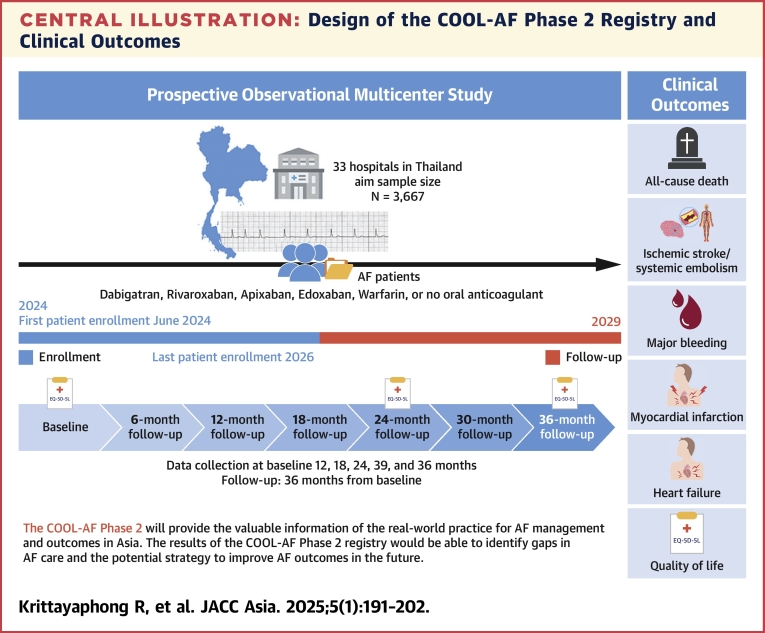
Design of the COOL-AF Phase 2 Registry and Clinical Outcomes The design of the COOL-AF (COhort of antithrOmbotic use and cLinical outcomes in patients with Atrial Fibrillation) Phase 2 registry, a prospective observational study involving 3,667 atrial fibrillation (AF) patients from 33 hospitals in Thailand. The timeline shows patient enrollment starting in June 2024, with follow-up assessments every 6 months. The study tracks key clinical outcomes, including all-cause death, ischemic stroke, systemic embolism, major bleeding, myocardial infarction, heart failure, and quality of life. The primary goal is to identify gaps in AF care and analyze real-world patterns of antithrombotic use to improve patient outcomes.

### Outcomes

Details of clinical outcomes are shown in Table 4. The definitions of main clinical outcomes are as follows:

• Ischemic stroke/TIA: acute onset of focal neurological deficit that persists more than 24 hours for ischemic stroke, and <24 hours for TIA. Imaging data from computed tomography brain scans or cardiac magnetic resonance are required to be uploaded into the web-based system.
• Major bleeding: International Society of Thrombosis and Haemostasis criteria.^19^ It is defined as intracranial hemorrhage, intraspinal hemorrhage, intraocular/retinal hemorrhage, intra-articular hemorrhage, or intramuscular hemorrhage with compartment syndrome, or any bleeding that leads to a reduction in hemoglobin of ≥2 mg/dL from baseline. Minor bleeding is defined as any bleeding that did not fulfill any of the major bleeding criteria.
• HF event: a hospital admission or a presentation of the patient for an urgent, unscheduled clinic, office or emergency department visit, with a primary diagnosis of HF. The clinical presentation may be new or worsening of HF symptoms, and there must be objective evidence of new or worsening HF, along with the initiation or intensification of treatment specifically for HF.^20^
• Myocardial infarction: Clinical syndrome characterized by evidence of myocardial necrosis in patients with a clinical setting consistent with acute myocardial ischemia.^21^

**Table 4:** Main Outcome Measures

Outcome Measures	Types
Clinical events	Ischemic stroke/TIA Systemic embolization Heart failure Myocardial infarction Death Cause of death (cardiovascular, noncardiovascular including details)
Bleeding events	
Severity	Major bleeding (ICH, non-ICH) Minor bleeding
Location	Intracranial Intraspinal Intraocular/retinal Retroperitoneal Intra-articular Pericardial Intramuscular with compartment syndrome Intramuscular without compartment syndrome Epistaxis Upper GI Lower GI Oral cavity Hemoptysis Prolonged menstrual Macroscopic hematuria Rectal Skin Subconjunctival
Outcome	Fatal Nonfatal
Health care used for bleeding event	Hospitalization (length of stay and cost) Transfusion
OAC persistence	Rate of discontinuation Duration of time on therapy Reasons for discontinuation
Hospital visits	Number of hospital visits related to AF
Patients treated with vitamin K antagonists	Number, date of INR monitoring Time in the therapeutic range Warfarin dose Type of test (central laboratory or POCT) Setting (OPD or IPD) Warfarin interruption and use of bridging

ICH = intracranial hemorrhage; IPD = inpatient department; OAC = oral anticoagulant; OPD = outpatient department; POCT = point of care test; TIA = transient ischemic attack.

The source documents are required to be uploaded into the web-based system for the verification of clinical outcomes. All source documents, including imaging for those who had a stroke or TIA, are sent to the adjudication committee for confirmation of the clinical outcome.

### Data management

Web-based data from the study sites will be submitted to the data management site or central site (Siriraj Hospital, Mahidol University, Bangkok, Thailand). Patients will be assigned a unique identification number. Any data that are transferred to the data management site will be anonymized for to protect personal identification information and confidentiality. Investigators at the data management site can review the data, verify data, and send queries to the study site for clarification. Site monitoring will be performed at every site to assess the data quality and to ensure that the study is conducted in accordance with Good Clinical Practice guidelines.

### Statistical analysis

Data analysis will be performed at the central site. Continuous variables with normal distribution will be presented as mean ± SD, and continuous variables with non-normal distribution will be presented as median and IQR. Categorical variables will be presented as absolute numbers and percentages. All statistical tests will be 2-tailed, and *P <* 0.05 will be regarded as significant. Moreover, if necessary, post hoc analyses will be conducted. The baseline characteristics of patients will be presented as mean ± SD, and frequency for continuous and categorical variables, respectively. The incidence rates of each clinical outcome will be presented as rates per 100 person-years with a Poisson 95% CI and a 2-sided *P* value. Univariable and multivariable analysis will be performed to identify factors that predict adverse clinical outcomes using the Cox proportional Hazard model. HRs and 95% CIs will be reported. The selection of variables in multivariable model will be based on the prior knowledge of the covariates that might impact the outcome and theory driven approaches that might be the predictors of each clinical outcome in combination with the results of the univariate analysis. Multivariable analysis using backward elimination with a *P* value <0.05 as the stopping criterion will be used. The association of the pattern of antithrombotic use and clinical outcome will be analyzed using the Cox model. For the warfarin group, the time in the therapeutic range (TTR) will be calculated using the Rosendaal method,^22^ The analysis related to warfarin will be performed with and without consideration of the TTR data.

#### Model development

We will follow the Transparent Reporting of a multivariable prediction model for Individual Prognosis or Diagnosis reporting guideline for the prediction model.^23^ The risk prediction model for the clinical outcomes of interest will be developed from the variables derived from the multivariable model. The probability of outcome at time A for each patient will be calculated by using the following equation, which is derived from Cox proportional hazards model:

$$P_{\text{Outcome}}\;\text{at}\;\text{time}\;A=1\;–\;S_{0}{\left(t\right)}^{\exp\left(\text{Prognostic}\;\text{Index}\right)}$$

where S_0_(t) is the average survival probability at time t (ie, at time A), and prognostic index is the sum of the predictor products and the coefficients obtained from the multivariable model.

#### Model validation

Model applicability assessment will be performed according to the Prediction model Risk Of Bias ASsessment Tool suggestions.^24^ Bootstrapping based on 100 to 1,000 bootstrap samples will be used to verify the fitted model. Calibration will be tested using the calibration slope based on the observed and predicted hazards of the outcome.^25^ The Harrell C-statistics will be used to measure model discrimination. C-statistics vary from 0 to 1, and a value close to 1 reflects a good prediction model.^26^ Receiver-operating characteristics curve will be used to calculate the area under curve and to compare C-statistics. The D-statistic assesses the discrimination ability of the fitted model for low- and high-risk patients.^26^ C- and D- statistics will also be calculated after the bootstrapping as an internal validation. The Brier score will be evaluated to assess the predictive ability of the model.^27^ Net reclassification index and integrated discrimination index will be calculated based on previously proposed methods to determine the influence of the predictive model on the reclassification of the study subjects.^28^ Decision-curve analysis will be used to estimate the clinical usefulness of the prediction models by assessing the ability to make better decisions with a model than without it.^29^

All statistics were performed using the SPSS statistical software version 18.0 (SPSS, Inc) and R version 3.6.3 (R Foundation for Statistical Computing).

### Sample size calculation

The sample size calculation for the predictive model development was based on the 10 events per variable concept, which suggested that we need at least 10 events per candidate variable.^30^ The results from COOL-AF phase 1 indicated that the number of candidate variables for death, SSE, and major bleeding is approximately 10. Using the rate of death, SSE, and major bleeding from COOL-AF phase 1,^3^ the number of patients needed for predictive model development was highest at 3,334 for SSE. If the rate of incomplete or loss follow-up is 10%, the sample size needed was 3,667. This sample size also covers the sample size needed to determine the rate of clinical outcomes (death, SSE, and major bleeding).

### Expected benefits

1. To ascertain the current usage patterns of antithrombotic medications in AF patients, particularly whether the use of DOACs has increased since the COOL-AF Phase 1 study and whether there have been improvements in the appropriate use of warfarin regarding INR levels and TTR.
2. To establish Thai guidelines for the appropriate antithrombotic medication use in AF management, aiming to enhance the quality and standardization of care.
3. To assess the rates of ischemic stroke and major bleeding in Thai populations with AF who are receiving warfarin and DOACs.
4. To foster a culture of collaboration and serve as a foundation for future joint research projects among institutions, hospitals, and cardiology societies.
5. To understand the economic implications of using DOACs in AF patients.
6. To advance the care of AF patients by reducing complications such as ischemic stroke and abnormal bleeding, with the goal of alleviating the economic burden associated with this disease.

### Trial status

The research funding for the COOL-AF Phase 2 study was approved by the Health System Research Institute and the Heart Association of Thailand. The study protocol was approved by the CREC (COA-CREC103/2023). The local Institutional Review Board has approved the study at one-half of the sites. The web-based system is in the final stage of preparation. The enrollment began in June 2024. Last patient recruitment is expected in June 2026, and the last follow-up visit is expected to be in June 2029.

## Discussion

The COOL-AF registry Phase 1 study received funding from the Health System Research Institute (HSRI) and the Heart Association of Thailand under the Royal Patronage. It is a prospective cohort study that enrolled 3,461 AF patients from 27 hospitals across Thailand. The enrollment period spanned from 2014 to 2017, and the participants were scheduled for follow-up visits every 6 months for up 3 years.^5^ The findings from the COOL-AF Phase 1 study have provided valuable insights into the use of anticoagulant medications and various aspects of managing AF patients in Thailand. Key findings from COOL-AF Phase 1 are summarized in Table 5.

**Table 5:** Key Findings From COOL-AF Phase 1

1. Thai AF patients had high risk of bleeding events including ICH^3^ 2. Even though the majority of patients received warfarin, the benefit of warfarin will be only in patients with good TTR (TTR >65%)^42^ 3. In patients with poor TTR (majority), giving warfarin had a negative net clinical benefit (benefit in stroke prevention less than risk of major bleeding) compared with not giving OAC at all (under submission) 4. Target INR of Thai AF patients is different from the international recommended INR. In patients with age <70 years, the target INR is 2.0-3.0, whereas in those age 70 years, the target INR was 1.5-3.0^43^ 5. DOAC is better than warfarin in Thai AF^44^^,^^45^ 6. Promotion of anticoagulation clinic is important to improve quality of warfarin use 7. Non-CV death is as important as CV death in AF patients^46^ 8. Promotion of holistic care of AF patients is essential to improve outcomes (appropriate OAC use, appropriate treatment of symptoms and comorbidities)^45^^,^^47^

CV = cardiovascular; other abbreviations as in Tables 1, 3, and 4.

### Contemporary OAC management in Asian population

The use of warfarin has been associated with frequent suboptimal INR levels, and there is an increasing trend in the use of DOACs.^5^^,^^31^^,^^32^ DOACs have a lower risk of abnormal bleeding compared with warfarin, highlighting the need for a study on the patterns of anticoagulant use in AF patients and their clinical outcomes (including all-cause mortality, ischemic stroke/TIA/systemic embolism, intracerebral hemorrhage, and major bleeding) when using DOACs compared with warfarin.^33^ Past data indicate that patients in Asia differ from those in Western countries.^11^ Therefore, it is crucial to investigate this data in the Thai context to determine whether there has been an increased use of DOACs or a change in clinical practice to achieve a TTR of >65% with warfarin. Additionally, monitoring the use of antiplatelet agents, such as aspirin, in Thai patients (especially those with coexisting coronary artery disease) is important because data from the COOL-AF Phase 1 trial does not support the use of antiplatelet agents in these patients.^34^

Currently, there are 4 types of DOACs available: dabigatran, rivaroxaban, apixaban, and edoxaban, which tend to be relatively expensive compared with warfarin. Therefore, it is necessary to conduct further studies on AF patients using DOACs to perform economic analyses of the complications related to ischemic stroke and to assess the cost-effectiveness of DOACs compared to warfarin.^35^

### Comparisons of previous and existing AF registries

Table 6 provides an overview of the selected AF registries. Among 8 AF registries listed, 2 were from Europe, 1 from the United States, 1 global, and 4 from Asia (including 1 from Asia-Pacific, 1 from Japan, 1 from India, and 1 from Thailand). The results of these registries demonstrate that Asians and non-Asians had some similarities and some discrepancies. An example of a similarity is that AF had high mortality and morbidity in both groups.^3^ The differences between Asians and non-Asians included the pattern of OAC use and a higher rate of major bleeding in Asians.^3^^,^^36^

**Table 6:** Overview of Selected Atrial Fibrillation Registries

	Population	Number of Patients	Follow-Up	Study Period	No. of Countries	No. of Sites	Quality-of-life Measures
ORBIT-AF^48^	Known and new AF (outpatient)	10,000	2 y	2009-2011	1 (United States)	∼200	AFEQT and ACTS
GARFIELD-AF^6^^,^^49^	New AF with at least 1 risk factor for stroke	28,628	2 y	2010-2016	35	>1,000	None
Fushimi AF^50^	Inpatient and outpatient (community based)	3,183	1 y	2011-2012	1 (Japan)	76	None
Euro Heart Survey on AF^51^	Known and new AF (inpatient and outpatient)	5,333	1 y	2003-2004	35	182	None
EORP-AF Registry^52^	Known and new AF (inpatient and outpatient)	3,119	1 y	2012-2013	9	67	None
APHRS AF^53^	Known and new AF (inpatient and outpatient)	4,664	1 y	2015-2017	5 (Hong Kong, Taiwan, Singapore, Japan, Korea)	52	None
Kerala AF^7^	Known and new AF (Inpatient and outpatient)	3,421	1 y	2016-2017	1 (India)	53	None
COOL-AF Phase 1^5^	Known and new AF (outpatient)	3,405	3 y	2014-2017	1 (Thailand)	27	EQ5D
COOL-AF Phase 2	Known and new AF (outpatient)	3,667	3 y	2024-2026	1 (Thailand)	33	EQ5D

APHRS AF = the Asia-Pacific Heart Rhythm Society Atrial Fibrillation registry; COOL-AF Phase 1 = The COhort of antithrombotic use and Optimal INR Level in patients with non-valvular Atrial Fibrillation in Thailand registry; COOL-AF Phase 2 = COhort of antithrOmbotic use and cLinical outcomes in patients with Atrial Fibrillation registry; EORP-AF = the EURObservational Research Programme Atrial Fibrillation registry; GARFIELD-AF = The Global Anticoagulant Registry in the FIELD–Atrial Fibrillation registry; ORBIT-AF = The Outcomes Registry for Better Informed Treatment of Atrial Fibrillation registry.

### Importance of AF registry in the era of changing OAC management pattern

Despite the availability of large clinical trials of DOACs, the real-world data is also important to demonstrate the use of OACs and other aspects of AF management in the clinical practice. It has been shown that both clinical trials and real-world evidence are complementary,^37^ and both clinical trial results and real-world data can guide decision making.^38^

### Cost and quality of life data

In the COOL-AF Phase 2 registry, we also collecting data on the cost and EQ-5D, which may help assess the cost burden in patients with AF. Additionally, the cost data and EQ-5D can be used for cost-utility analysis to compare 2 treatment groups such as warfarin vs DOACs.

### Future directions

One area of interest for our team is the AF management in rural area. It has been shown that AF management between urban and rural regions may be different.^39^ The mortality rate is higher in AF patients among AF patients in rural areas.^40^ Both physician and patient perceptions of AF care in these regions reveal many social and structural barriers to effective management.^41^ We plan to explore the data of AF management in small rural hospitals and investigate opportunities to improve care and outcome for patients in these area.

### Study limitations

First, this is a prospective multicenter study conducted in Thailand. However, most of the hospitals participating in this study are large hospitals and may not fully represent AF data for the entire nation because the data from smaller hospitals are lacking. Second, the majority of patients in our study are on warfarin as their OAC choice. Despite the shift in in clinical practice in OAC choice by moving toward the greater use of DOACs, DOACs are currently not included in the national drug list. We expect that the data of COOL-AF Phase 2 will provide contemporary insights into OAC management in our country. We estimate that DOACs may be used in approximately one-half of patients requiring OAC therapy.

### Study strengths

First, this is a multicenter study with prospective data collection. Second, we are collecting data every 6 months for up 3 years. This data collection plan will allow us to have granular data to assess the time-updated models to evaluate the impact of changes in management and clinical data over time on the clinical outcome. Third, we require the source document data to be upload into the web system for all clinical outcomes. The adjudication team will confirm each outcome.

## Conclusions

AF remains a major cardiovascular problem especially in Asian countries. Despite the Asian population’s use of warfarin and DOACs having a higher risk of ICH compared with non-Asians, OAC management remains a major issue in many low- and middle-income Asian countries because of the cost of DOACs. Results of COOL-AF Phase 1 confirmed that the Asian population is a high-risk group and that appropriate management according to the current guideline is required to improve outcomes. The results of the COOL-AF Phase 2 will provide valuable data for the contemporary AF management, reflecting the changing trend of DOACs and in the increasing implementation of practice guidelines.

## Funding Support and Author Disclosures

This study was funded by grants from the Health Systems Research Institute (grant number 67-057) and the Heart Association of Thailand under the Royal Patronage of H.M. the King. None of the aforementioned funding sources influenced any aspect of this study or the authors' decision to submit this paper for publication. Dr Lip has served as a consultant and speaker for Bristol Myers Squibb/Pfizer, Boehringer Ingelheim, Daiichi-Sankyo, and Anthos; no fees are received personally. All other authors have reported that they have no relationships relevant to the contents of this study to disclose.
