# Supplementary material for: The COOL-AF Phase 2 Registry: COhort of Antithrombotic Use and Clinical Outcomes in Atrial Fibrillation Patients
Source: JACC Asia. 2025 Jan 21;5(1):191–202. doi: 10.1016/j.jacasi.2024.10.027 (PMC11782006; doi:10.1016/j.jacasi.2024.10.027)
Supplement: Supplementary file 1 [file mmc1.docx]

The COOL-AF Phase 2 Registry

COhort of Antithrombotic Use and Clinical Outcomes in

Atrial Fibrillation Patients

List of COOL-AF phase 2 investigators:

Bhumibol Adulyadej Hospital: Panyapat Jiampo; Buddhachinaraj Hospital: Tomorn

Thongsri; Central Chest Institute of Thailand: Komsing Methavigul; Charoen Krung Pracha Rak Hospital: Natchayathipk

Kittichamroen; Chiangrai Prachanukroh Hospital: Wattana Wongtheptien; Chonburi Hospital: Chanadda Rutnuntamongkon;

Faculty of Medicine, HRH Princess Maha Chakri Sirindhorn Medical Center (MSMC) Srinakharinwirot University: Nattapun

Rattanajaruskul; Faculty of Medicine, Chiang Mai University: Arintaya Phrommintikul; Faculty of Medicine, Chulalongkorn

University: Voravut Rungpradubvong; Faculty of Medicine, Khon Kaen University: Pattarapong Makarawate; Faculty of

Medicine, Naresuan University: Nattakorn Songsirisuk; Faculty of Medicine, Prince of Songkla University: Ply Chichareon;

Faculty of Medicine, Ramathibodi Hospital, Mahidol University: Sirin Apiyasawat; Faculty of Medicine, Siriraj Hospital, Mahidol

University: Arjbordin Winijkul; Faculty of Medicine, Siriraj Hospital, Mahidol University: Rungroj Krittayaphong; Faculty of

Medicine, Thammasat University, Rangsit Campus: Kumpol Chintanavilas; Faculty of Medicine, Vajira Hospital, Navamindradhiraj

University: Teetouch Ananwattanasuk; Golden Jubilee Medical Center: Pantitra Pipatsawadwut; Lampang Hospital:

Thanita Boonyapiphat; Maharat Nakorn Ratchasima Hospital: Keerapa Phusunti; Nakornping Hospital: Khanchai Siriwattana;

Phramongkutklao College of Medicine: Thoranis Chantrarat; Police General Hospital: Thanapol Olarnrachin; Prapokklao Hospital

(Chanthaburi): Wiwat Kanjanarutjawiwat; Queen Savang Vadhana Memorial Hospital: Sakaorat Kornbongkotmas; Rajavithi

Hospital: Apichai Pokawattana; Ratchaburi Hospital: Thunyarat Chaipruckmalakarn; Sawanpracharak Hospital: Thitisan Luengsupaboon;

Sunpasitthiprasong Hospital: Pathompong Kumpamool; Suratthani Hospital: Suchart Arunsiriwattana; Surin Hospital:

Thaworn Choochunklin; Udonthani Hospital: Anuchit Wongphen; Vachira Phuket Hospital: Nongnuch Weerapakorn.
